# Antimicrobial Potential and Cytotoxicity of Silver Nanoparticles Phytosynthesized by Pomegranate Peel Extract

**DOI:** 10.3390/antibiotics7030051

**Published:** 2018-06-26

**Authors:** Renan Aparecido Fernandes, Andresa Aparecida Berretta, Elina Cassia Torres, Andrei Felipe Moreira Buszinski, Gabriela Lopes Fernandes, Carla Corrêa Mendes-Gouvêa, Francisco Nunes de Souza-Neto, Luiz Fernando Gorup, Emerson Rodrigues de Camargo, Debora Barros Barbosa

**Affiliations:** 1 Department of Dentistry, University Center of Adamantina (UNIFAI), Adamantina 17800-000, São Paulo, Brazil; renanfernandes@fai.com.br; 2 Department of Dental Materials and Prosthodontics, São Paulo State University (UNESP), School of Dentistry, Araçatuba 16015-050, São Paulo, Brazil; fernandesgabriela@hotmail.com (G.L.F.); carla_cmendes@hotmail.com (C.C.M.-G.); 3 Laboratory of Research, Development & Innovation, Apis Flora Industrial e Comercial Ltda., Ribeirão Preto 14020-670, São Paulo, Brazil; andresa.berretta@apisflora.com.br (A.A.B.); elinacassia@hotmail.com (E.C.T.); andrei.buszinski@apisflora.com.br (A.F.M.B.); 4 Department of Chemistry, Federal University of São Carlos, São Carlos 13565-905, São Paulo, Brazil; francisconsn29@gmail.com (F.N.S.-N.); lfgorup@gmail.com (L.F.G.); camargo@ufscar.br (E.R.C.); 5 FACET-Department of Chemistry, Federal University of Grande Dourados, Dourados 79804-970, Mato Grosso do Sul, Brazil

**Keywords:** silver, nanoparticles, *Candida albicans*, *Staphylococcus aureus*, herbal medicine, Punicaceae

## Abstract

The phytosynthesis of metal nanoparticles is nowadays attracting the increased attention of researchers and is much needed given the worldwide matter related to environmental contamination. The antimicrobial activity of colloidal and spray formulation of silver nanoparticles (AgNPs) synthesized by pomegranate peel extract against *Candida albicans* and *Staphylococcus aureus*, and their cytotoxicity in mammalian cells were tested in the present study. Dry matter, pH, total phenolics, and ellagic acid in the extract were determined. Then, AgNPs were phytosynthesized and characterized by X-ray diffraction, electron transmission microscopy, dynamic light scattering, zeta potential, and Ag^+^ dosage. Spray formulations and respective chemical-AgNP controls were prepared and tested. The peel extract reduced more than 99% of Ag^+^, and produced nanoparticles with irregular forms and an 89-nm mean size. All AgNP presented antimicrobial activity, and the spray formulation of green-AgNP increased by 255 and 4 times the effectiveness against *S. aureus* and *C. albicans*, respectively. The cytotoxicity of colloidal and spray green-AgNP was expressively lower than the respective chemical controls. Pomegranate peel extract produced stable AgNP with antimicrobial action and low cytotoxicity, stimulating its use in the biomedical field.

## 1. Introduction

Recently, a state of alert on a topic that affects people globally, antimicrobial resistance, has received much attention. This has led to the deaths of more than 700,000 people a year worldwide and this number has risen every year [1]. It is estimated that there will be a reduction in the world population of 11–444 million people in 2050 if antimicrobial resistance is not bypassed [1].

As an alternative against antimicrobial resistance, one approach gaining in strength is the use of inorganic particles at the nanoscale. The most prominent metals in the group of inorganic nanoparticles are copper, zinc, titanium, magnesium, gold, and silver [2,3,4]. In this context, silver nanoparticles have been the most exploited as they have a wide range of toxicity against several microorganisms such as *Staphylococcus aureus*, *Escherichia coli*, *Candida albicans*, and others [5].

The incorporation and use of silver nanoparticles has been observed in sundry sectors, for instance, in the food industry as an attempt to produce packaging with antimicrobial activity [6]. Its use in the area of cosmetics has also received prominence, as has its use in housecleaning, antiseptics, sunscreens, soap, and shampoo [7,8,9] as well as in textile manufacturing [10].

Considering the synthesis of silver nanoparticles, many routes have been presented such as electrochemical [11], radiation [12], photochemistry [13], and by biological methods [14]. Phytochemical synthesis has been noteworthy since the use of chemical compounds may result in undesirable toxic effects not only for the human organism but also for the environment. Its effectiveness in the production of silver nanoparticles has been demonstrated by the use of compounds of different plants in the ion reduction, being characterized as rapid, low cost, and environmentally friendly synthesis [15]. Furthermore, green-silver nanoparticles are usually less cytotoxic when compared to those reduced by conventional chemical agents [16]. It is believed that silver nanoparticles reduced by plant extracts do not carry on their surface chemical compounds used for the reduction and stabilization of chemically produced silver nanoparticles that are toxic to human cells. It is still believed that the phytochemicals present in the extracts are carried on the surface of the silver nanoparticles, reducing their cytotoxic effect, aside from presenting different forms of chemically produced silver nanoparticles [16]. Important aspects in green-synthesis should be taken into account including the choice of plant to be used, being the plants which grow in different regions of the world more eligible for this [16]. The previously known potential of the plant including antioxidant, anti-inflammatory, and antimicrobial such as the case of *Punica granatum* (pomegranate) should also be considered [17,18,19]. Some studies have also used *Punica granatum* to reduce silver ions to silver nanoparticles [19,20,21]. Silver nanoparticles were green-synthesized and showed significantly lower cytotoxicity when compared to the silver nanoparticles synthesized by a chemical pathway. This fact has stimulated the search for the use of reduced silver nanoparticles by means of plant extracts for biological purposes such as the treatment of contagious infectious diseases, especially those in need of topical treatment.

Thus, taking together the benefits of pomegranate and the antimicrobial applicability of silver nanoparticles, the present study aimed to synthesize silver nanoparticles using pomegranate peel extract, and to produce spray formulations containing the previously green-synthesized silver nanoparticles. Their antimicrobial activity against *Staphylococcus aureus* and *Candida albicans*, and their cytotoxicity effect on fibroblast cells were investigated.

## 2. Results

### 2.1. Characterization of Peel Extract, Silver Nanoparticles and Formulations

The pH and the dry matter of the peel extract obtained by maceration followed by percolation were 3.13 and 86.39 (±0.96) % *w/w*, and the total phenolics expressed in gallic acid and the ellagic acid were 392.0 (±9) and 3.64 (±0.03) mg/g, respectively.

**Figure 1:**
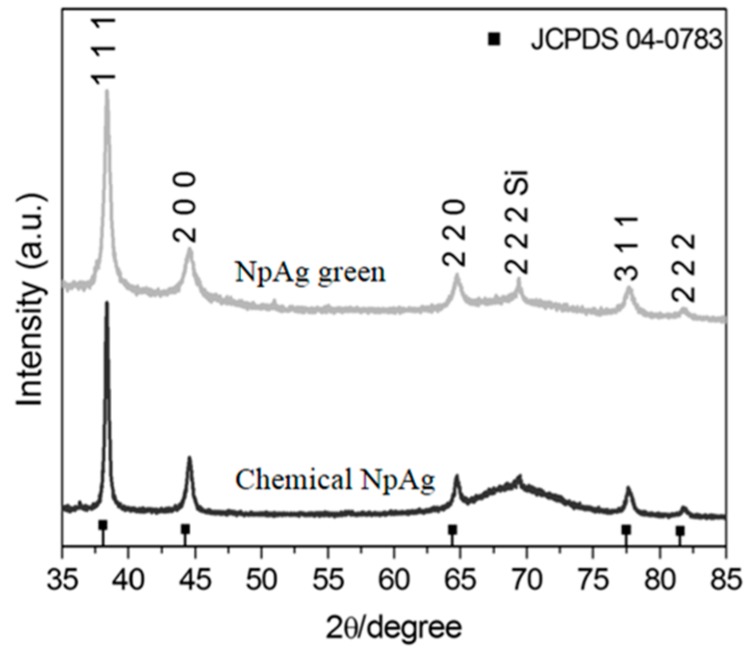
X-ray diffraction (XRD) of the green and chemical silver nanoparticles.

**Figure 2:**
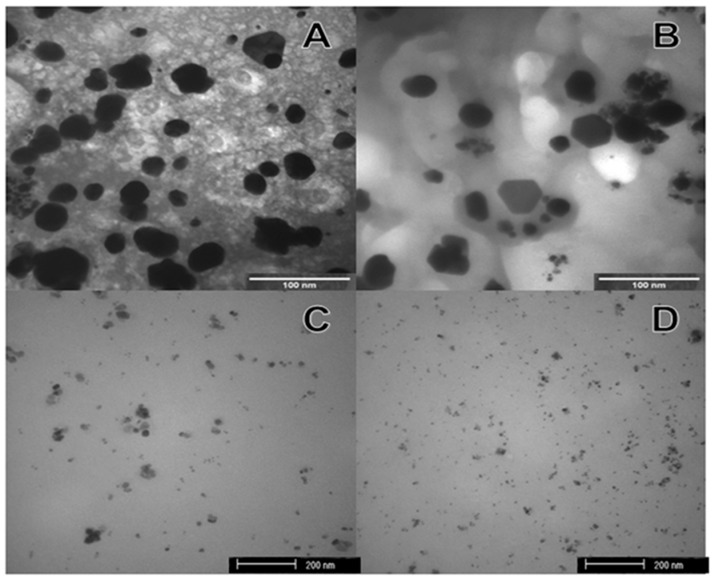
Images of transmission electron microscopy (TEM): (**A**) Green silver nanoparticles; (**B**) Silver nanoparticles green formulation; (**C**) Chemical silver nanoparticles; (**D**) Silver nanoparticles chemical formulation.

The formation of silver nanoparticles was confirmed by comparing the XRD patterns and the corresponding standard patterns of cubic of silver nanoparticles (Figure 1), according to the diffraction standard (JCPDS file No. 04-0783). The reflection peak (2 2 2) is characteristic of the substrate (Si), where silver particles were deposited as a thin film. TEM images (Figure 2) showed different forms and sizes of silver nanoparticles produced by green and conventional chemical routes as well as in their respective formulations. In general, green-synthesis produced particles with a larger size than those obtained by conventional synthesis. Dynamic Laser Scanning (DLS) analyses of the formulations prepared with green or conventional silver nanoparticles demonstrated different particle sizes, being the mean values of 89 ± 21 and 19 ± 4 nm for the green and conventional formulation, respectively. The values of zeta potential of green and conventional silver nanoparticles were lower than −30 mV (−46.2 ± 6.06 mV green, and −67.5 ± 3.69 mV conventional), indicating the stability of both colloidal silver nanoparticles.

**Figure 3:**
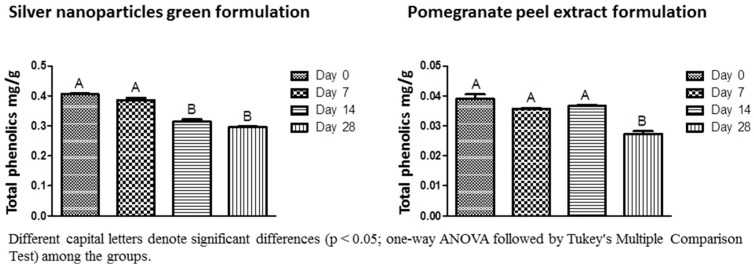
Total phenolics concentration for the silver nanoparticles green formulation and pomegranate peel extract formulation in different periods. Different capital letters denote significant difference (*p* < 0.05; one-way ANOVA followed by Tukey’s multiple comparison test) among the groups.

Almost 100% of the Ag^+^ ions coming from AgNO_3_ were reduced by the pomegranate peel extract (99.89%) and sodium citrate (99.51%). However, in the spray formulation containing chemical-silver nanoparticles, the percentage of reduction was diminished to 68.18% although the formulation maintained stable regarding Ag^+^ ions concentration for 28 days (Table 1). Zeta potential data confirmed the stability of the spray formulations regardless of the method used to obtain the silver nanoparticles (Table 2). The total phenolics in the spray formulations with or without silver nanoparticles were quantified at 0, 7, 14, and 28 days after having been prepared (Figure 3), and it has been significantly reduced in the green-synthesized silver nanoparticle formulation after 14 days with values ranging from 0.405 to 0.295 mg/g.

### 2.2. Antimicrobial Activity

The antimicrobial activity expressed as MIC values of silver nanoparticles and pomegranate peel extract (µg/mL) (Table 1) was, in general, considerably lower for the spray formulations than the active inputs regardless of the microorganisms tested. MIC values against *C. albicans* for active inputs and spray formulations were 781 and 0.18 for the peel extract, 68.75 and 16.87 for the green-, and 0.25 and 1.12 for the chemical-silver nanoparticles. While for *S. aureus*, the values were 391 and 0.37, 67.5, and 0.26, and 0.5 and 0.56 for pomegranate peel extract, green-, and chemical-silver nanoparticles in the active inputs and spray formulations, respectively. In addition, different conditions of humidity and temperature did not affect the effectiveness of the spray formulations against both microorganisms.

### 2.3. Cytotoxicity

**Figure 4:**
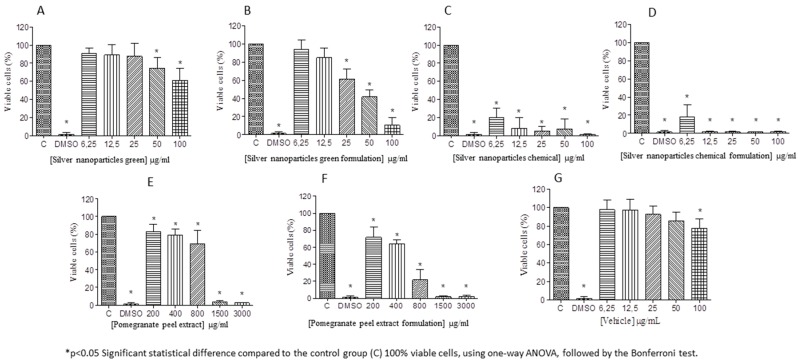
Cytotoxicity evaluation of respective active input (green and chemical silver nanoparticles and pomegranate peel extract), their respective formulations, and the vehicle (compounds of spray-formulation without the active inputs). (**A**) Silver nanoparticles green; (**B**) Silver nanoparticles green formulation; (**C**) Silver nanoparticles chemical; (**D**) Silver nanoparticles chemical formulation; (**E**) Pomegranate peel extract; (**F**) Pomegranate peel extract formulation and (**G**) Vehicle.

Figure 4 shows the fibroblast L929 cells viability in view of different concentrations of silver nanoparticles (green and conventional route). Green silver nanoparticles presented lower cytotoxicity than conventional ones. A dosage of 50 µg/mL was necessary to initiate the toxicity, but the cell viability was nearly 80%, while conventional-silver nanoparticles were quite toxic at very low concentration (6.25 µg/mL) and was similar to the negative control (DMSO) with viability lower than 20%. Furthermore, the addition of the reagents to prepare the formulations did not interfere in the toxicity of the conventional-silver nanoparticles, whereas the cytotoxicity for the green-silver nanoparticles formulation as well as for the extract formulation was considerably increased. 

## 3. Discussion

For future reproducibility of the experiment, the extract obtained by maceration followed by percolation was duly characterized in relation to dry matter, total phenolics content, ellagic acid, and pH. Total phenolics were determined only in samples that contained the pomegranate peel extract, and then the chemical formulation did not present any phenolic content in its composition. Polyphenols are effective hydrogen donors and are correlated to the number and position of hydroxyl groups and conjugations as well as the presence of donor electrons in the aromatic ring B, because of the ability of this aromatic ring to withstand the electron depletion located in the π electron system [22]. The antimicrobial activity of various polyphenols and plant extracts have been evaluated in pharmaceutical and food studies [23,24]. Some phenolic compounds present in sage, rosemary, thyme, hops, coriander, tea, cloves, and basil are known to exhibit antimicrobial effects against foodborne pathogens. Their mechanisms of action need to be further elucidated, and might be due to a plethora of phenolic compounds present in a very single plant extract. Furthermore, as the bioactive compounds in the extract presented antioxidant and anti-inflammatory activities, the antimicrobial potential of the pomegranate peel extract in the in vivo trials could show better results, and should be strongly stimulated in further studies. Regarding the multi conceptual nature of the term antioxidant and bringing it into the context of this study, some polyphenols present in low concentrations could prevent or reduce the extent of oxidative damage in mammalian cells. Taken together, these natural biomolecules could indirectly protect the cells and reduce the cytotoxicity of silver nanoparticles. 

The correct selection of the plant and the standardization of the methods to obtain the extracts to be used as reducing or capping agent in the nanosynthesis of metal particles should be preponderant when the green process is elected for the production of products in large scale. Additionally, a plethora of plants used in the phytosynthesis of metal nanoparticles [25,26,27] and the lack of information of the extraction techniques used in the articles has hindered the comparison of the present results with those found in the literature. For instance, different values and methods of total phenolics quantification can be observed in the literature as described by Kalaycioglu et al. (2017) [28]. Similarly, other factors can interfere in the evaluation and comparisons of the extracts such as the chemical and genotypic composition of the plant, the variety and the soil type, the place of the plant origin, the harvest season, maturation method, aside from the solvent and the process used for the obtention of the pomegranate extract, among others [29].

Scanning electron microscopy (SEM) and transmission electron microscopy (TEM) images showed the smallest particles obtained by conventional chemical synthesis, and DLS data confirmed these findings with mean sizes of 89 and 19 nm for green and chemical nanoparticles, respectively. The fission of colloidal particles of different sizes and shapes may be related to additives (salts, polymers), solvent properties (boiling temperature, affinity with created surfaces), the addition of nucleation, among others [30]. The reagents used in the chemical synthesis would produce particles with more predictable characteristics than the several substances and compounds present in the plant extract and used in the phytosynthesis route, which would interfere with the size and form of the nanoparticles and make phytosynthesis a challenge in controlling the reaction process and the morphological aspects of the particles. Moreover, the presence of different bioactive substances in the extract would reduce only a fraction of the silver ions present in the solution. The remaining silver ions would form other nuclei and further the growth of the previously formed silver nanoparticles [31]. This process is called Ostwald Ripening, where the largest particles consume the smaller ones and grow larger, where the dissolution of the smaller ones and deposition of ions on the surface of larger ones occur [32].

Almost 100% of ions reduction was observed for both synthesis routes. However, when the chemical silver nanoparticles were added to the formulation, a dissociation of ions from nearly 30% was observed when compared to chemical silver nanoparticles alone. This fact could be due to the presence of the components as carboxymethylcellulose and propylene glycol in the spray formulation which possibly favored the silver ion dissociation into the system [33]. The presence of oxygen or ligands for Ag^+^ in the formulations may increase the dissolution rate of AgNP and lead to increased dissolution through the formation of Ag^+^ complexes [34]. Ag^+^ in solution will interact with various ions and molecules that are present in aqueous media. Important ligands to be considered for Ag^+^ are sulfide and organic ligands such as the carboxylic acids group which are used as Ag coatings (e.g., citrate, lactate). Carboxyl ligands such as carboxymethylcellulose strongly bind Ag^+^, which may affect the dissolution of AgNP and the bioavailability of Ag^+^ [35]. 

Furthermore, the size of the Ag in the NP affects the extent and kinetics of the AgNP dissolution as the smallest nanoparticles dissolve faster and to a greater extent [36]. This would explain the difference in the dissolution of the nanoparticles in the formulations. Their dissolution is of high relevance for the possible toxic effects of AgNP as Ag^+^ appears in many cases to determine their toxicity [37]. This fact was not observed when green-synthesis was carried out. This could be related to several compounds present in the extract which would readily react with the released silver ions, or the encapsulation of the silver nanoparticles promoted by those phytocompounds may have avoided the silver ions dissociation from the silver nanoparticles and its release to the solution. 

Zeta potential test demonstrated the stability of the silver nanoparticles, most notedly in the spray formulations. Electrical charges on the surface of the nanoparticles prevent agglomeration, and thus afford the stability of the nanoparticles [38,39]. Indeed, silver nanoparticles and spray formulations presented a mean of 70 mV, which indicates their high stability of silver nanoparticles [40].

Antimicrobial results are also promising for the silver nanoparticles as well as the pomegranate extract obtained. The formulations notably showed better results when compared with the input active only. This fact could be explained for the proper dispersion of the active inputs (silver nanoparticles and pomegranate peel extract) in the spray formulation. Additionally, a synergistic effect could have occurred between those active inputs and the methylparaben present in the formulation. In the literature, studies with an antimicrobial effect of pomegranate extract were conducted against *Staphylococcus aureus*, *Enterobacter aerogene*, *Salmonella typhi*, and *Klebsiella pneumonia* [41]. The MIC values obtained in this study for pomegranate extract were in accordance with Bakkiyaraj et al. (2013) [42] for both the microorganisms studied, and a difference was observed in *C. albicans*, but this fact may be explained by the difference between the *C. albicans* strains used in the studies. 

Chemical-silver nanoparticles, in formulation or not, produced MIC values against *S. aureus* about 10-fold lower than those produced by Prema et al. (2017) [33] (60 µg/mL), who also produced silver nanoparticles stabilized with CMC. Indeed, the antimicrobial activity of chemical silver nanoparticles was also determined by Monteiro et al. (2011) [43] with MIC values for *C. albicans* (0.5 µg/mL) in accordance with this present study.

Noteworthy is the difference found in the present study in respect of cytotoxicity between the chemical and green routes to obtain silver nanoparticles. Studies have shown that silver nanoparticles produced with *Protium serratum* and *Nyctanthes arbortristis* extracts were biocompatible when tested in L929 fibroblasts [44,45]. It is believed that what makes the silver nanoparticle toxic to human cells is the type of reducing agent used such as sodium citrate or sodium borohydride [46]. Even in conventional syntheses of silver nanoparticles, reagents are used that prevent the aggregation of these nanoparticles [47], which may further favor their cytotoxicity. 

In the case of phytosynthesis of metal nanoparticles, plant extracts, aside from acting as reducing agents, would act to stabilize the particles against dissolution, hence reducing the toxicity of the silver nanoparticles solution. Furthermore, it is possible that some compounds in the extracts may have a synergistic effect with the silver nanoparticles [48], making them less toxic to human cells. Furthermore, extracts of *Punica granatum* have exhibited antioxidant [49] and anti-inflammatory [50] activity, and may have contributed to reducing the cytotoxicity of green- in comparison with chemical-silver nanoparticles. 

In general, the stability assay (silver ions dosage, zeta potential, and antimicrobial activity) showed a high stabilizing capacity of the formulations. However, the spray formulations of green silver nanoparticles and pomegranate peel extract showed a significant reduction in the content of total phenolics in 14 and 28 days. The decrease in the content of total phenolics may have occurred due to the temperature variations inherent in the stability test, as occurred in the study of [51] where the temperature affected the total phenolics content in the roselle-mango juice blends. Moreover, in formulations containing green-silver nanoparticles, the components of the extract may have been degraded or associated with the nanoparticles, explaining the faster decrease of the total phenolics content when compared to the pomegranate extract formulation. Interestingly, ion dosage, zeta potential, and antimicrobial activity were not affected by different conditions of temperature, time, and humidity of the stability test. 

Altogether, the reported results suggest that the plant extract mediated syntheses of AgNP showed a pronounced lower cytotoxic effect in mouse fibroblast cells (L929) than the syntheses of AgNP by the chemical method. Of note is the implication that different sizes between the green- chemical-AgNP as well as the expected impurities sedimented on both obtained nanoparticles could have had on their toxicity. Although it is quite tricky to obtain AgNP with a well-defined form and size and prevent the particles aggregation [52], it is of importance to complement and support our findings, then strongly recommend an eco-friendly approach to producing green-AgNP and prototype wound-care sprays containing these particles. 

## 4. Materials and Methods

### 4.1. Plant Material and Preparation of Pomegranate Peel Extract

Pomegranate samples were collected from a crop cultivated in Eixo (21°08′01′′ S, 51°06′06′′ W), Mirandópolis, São Paulo, Brazil, during May 2015. Pomegranate peels were separated and stove-dried at 50 °C, ground, and sieved to a granulometry lower than 2 mm. Peels were submitted to alcohol extraction using 70% ethanol by maceration, followed by the percolation process [53]. The extract was characterized in relation to pH, dry matter, and total phenolics expressed as gallic acid. The chemical marker of pomegranate, ellagic acid, was also identified and quantified.

#### 4.1.1. Determination of Total Phenolics, pH, and Dry Matter

To determine the total phenolics, an analytical curve of gallic acid (Sigma-Aldrich Chemical Co., St. Louis, MO, USA) was carried out [54]. All extracts obtained and the standard solution of gallic acid were prepared in 50 mL volumetric flasks using water as the solvent. The samples were homogenized and, the flasks were brought to the ultrasonic bath for 30 min. A 0.5 mL aliquot was transferred to another 50 mL flask where 2.5 mL of Folin-Denis reagent (Qhemis-High Purity, Hexis, São Paulo, Brazil) and 5.0 mL of 29% sodium carbonate (Cinética, São Paulo, Brazil) were added. The samples were protected from light and the readings were performed after 30 min in a UV-Vis spectrophotometer at 760 nm [53]. The pH was measured direct from a solution of 1% extract, using a pH kit (Merck KGaA, Darmstadt, Germany) and dry matter was calculated after drying on a sample stove at 105 °C and was expressed in percentage *w*/*w*. All data were analyzed in triplicate.

#### 4.1.2. Determination of the Ellagic Acid Content

A Shimadzu liquid chromatograph and a Shimpack ODS C18 (Shimadzu Corporation, Kyoto, Japan) reverse phase column (100 mm × 2.6 mm) were used to determine the ellagic acid content by high performance liquid chromatography (HPLC). Analytical conditions were optimized based on de Sousa et al. (2007) [55] with modifications. As the mobile phase, HPLC grade methanol and a 2% aqueous acetic acid solution with gradient elution (0–7 min, 20–72.5% v/v methanol, 7–7.5 min, 72.5–95% v/v methanol, 7.5–8.5 min 95% v/v methanol, 8.5–9 min 95–20% v/v methanol, 9–10 min 20% v/v methanol) were used. The flow rate was 1.0 mL/min, and the separation was achieved at 25 °C. The injection volume was 5 μL and the wavelength used was 254 nm. Peaks were determined by comparison with an authenticated ellagic acid standard. Briefly, the sample was transferred to a 20 mL volumetric flask which was diluted with HPLC grade methanol. Extraction was undertaken using a vortex for 5 min and ultrasonic bath for 1 h. For the extracts, samples were transferred to volumetric flasks of 10 mL, using methanol HPLC as the solvent. All samples were vortexed for 5 min and sonicated for 30 min. Samples were filtered through 0.45 μm filter. All samples were prepared in triplicate.

### 4.2. Synthesis of Green-Silver Nanoparticles

The protocols described by Gorup et al. (2011) [56] and Das et al. 2015 [57] with modifications were used to produce silver nanoparticles. Briefly, 3.5% of carboxymethylcellulose (CMC) (Labsynth, Diadema, Brazil), 20% of propylene glycol (PG) (Labsynth, Diadema, Brazil), 100 mM of silver nitrate (SN) (Merck KGaA, Darmstadt, Germany), pomegranate peel extract at 30 mg/mL, and water to make up 100% of the samples were used. Silver nanoparticles were not purified relative to the excess reagents. The reaction was carried out at 50 °C for 12 min, and it was selected based on previous results. 

### 4.3. Synthesis of Chemical-Silver Nanoparticles

Chemical-silver nanoparticles were produced according to Gorup et al. [53]. AgNO_3_ (Merck KGaA, Darmstadt, Hesse, Germany) was dissolved in water, and brought to boiling at 90 °C. After 2 min of boiling, an aqueous solution of sodium citrate (Na_3_C_6_H_5_O_7_) (Merck KGaA, Darmstadt, Hesse, Germany) was added, and kept boiling for another 6 min until the solution reached a yellow amber color. The stoichiometric ratio was 1:3, respectively for AgNO_3_ and Na_3_C_6_H_5_O_7_. Silver nanoparticles were not purified relative to the excess reagents.

### 4.4. Preparation of the Spray Formulations

The reagents used were CMC (Labsynth, Diadema, Brazil), PG (Labsynth, Diadema, Brazil), and methylparaben (Labsynth, Diadema, Brazil) in a proportion of 0.1%, 7%, and 0.1%, respectively. The active inputs (green- or chemical-silver nanoparticles and pomegranate peel extract) concentrations were based on the minimum inhibitory concentration and cytotoxicity. Therefore, the final concentrations of active inputs in the spray formulations were: 337.5 µg/mL of green-silver nanoparticles, 5.55 µg/mL chemical-silver nanoparticles, and 94 µg/mL of crude peel extract dry matter. 

### 4.5. Characterization of the Silver Nanoparticles and the Spray Formulations

#### 4.5.1. X-ray Diffraction (XRD), Dynamic Light Scattering (DLS), and Zeta Potential Analysis

A Shimadzu XRD diffractometer with a Cu Kα radiation operating at 30 kV and 30 mA and 2θ range from 35° to 85° with step scan of 0.02° and scan speed 0.2°·min^−1^ was used to perform XRD analysis. To collect silver nanoparticles patterns, the nanoparticles were deposited on the surface of a silicon substrate (Si) by dripping the aqueous colloidal dispersion on the substrate at room temperature until the solvent had evaporated.

DLS experiments were performed at room temperature and at a fixed angle of 173° on a Zetasizer Nano ZS (Malvern Instruments Ltd., Malvern, UK) equipped with 50 mW 533 nm laser and a digital auto correlator. The number-average values obtained were compared to the size distributions of the silver nanoparticles. For the zeta potential test a Zetasizer (Malvern instruments, Malvern, UK) with an MPT-2 titrator was used. Aliquots from each test suspension were obtained to conduct zeta potential, and mean values were obtained from three independent measurements.

#### 4.5.2. TEM Analyzes

The nanocompounds morphology was characterized by TEM images in a Jeol JEM-100 CXII (JEOL USA Inc., Peabody, St. Louis, MO, USA) microscope equipped with Hamamatsu ORCA-HR digital camera.

### 4.6. Silver Ions Dosage

The dosages of free silver ions (Ag^+^) present in the compounds and spray formulations were performed to observe if the total amount of Ag added in the synthesis reaction was successfully reduced. A specific electrode 9616 BNWP (Thermo Scientific, Beverly, MA, USA) coupled to an ion analyzer (Orion 720 A^+^, Thermo Scientific, Beverly, MA, USA) was used. A 1000 µg Ag/mL standard was prepared by adding 1.57 g of AgNO3 to 1 L of deionized water. The combined electrode was calibrated with standards containing 6.25 to 100 µg Ag/mL to achieve equivalent silver concentrations in the compounds. A silver ionic strength adjuster solution (ISA, Cat. No. 940011) that provided a constant background ionic strength was used (1 mL of each sample/standard: 0.02 mL ISA).

### 4.7. Stability Test of the Spray Formulations

The spray formulations were submitted to a stability test with controlled conditions of temperature and time. This test was based on Anvisa protocols (Cosmetics stability guide ISBN 85-88233-15-0; Copyright^©^ Anvisa, 2005) and the guide to stability studies (Ordinance No. 593 of 25 August 2000). Briefly, samples of each spray formulation were submitted to alternating cycles of temperature daily ranging from 40 to −5 °C for 28 days. The tests selected to evaluate the stability of the samples were ion dosage, total phenolics content, zeta potential, and minimal inhibitory concentration (MIC). All tests were done in the same conditions as described before, and were carried out at 0, 7, 14, and 28 days.

### 4.8. Antimicrobial Activity of the Silver Nanoparticles and the Spray Formulations

Minimal inhibitory concentration of the silver nanoparticles samples were determined following the instructions of the Clinical Laboratory Standards Institute with some modifications. The samples were first diluted in water and subsequently in culture medium specific for each microorganism, Mueller Hinton broth (BD Difco, Franklin Lakes, USA) for *Staphylococcus aureus* (ATCC 25923), and RPMI (Sigma-Aldrich, St. Louis, MO, USA) for *Candida albicans* SC 5314) [58]. The microorganisms were adjusted to 5 × 10^5^ cells/mL for *S. aureus* and 5 × 10^3^ cells/mL for *C. albicans*, and the plates were incubated for 24 h and 48 h in aerobiosis at 37 °C for *S. aureus* and *C. albicans*, respectively. After incubation, the plates were visually read. The assays were performed in triplicate.

### 4.9. Cytotoxicity Analysis

For the evaluation of cytotoxicity, fibroblast cells of the L929 lineage were used. Cells were cultured in DMEM culture supplemented with 10% fetal bovine serum (FBS), penicillin G (100 U/mL) (Gibco^®,^ Carlsbad, USA), streptomycin (100 μg/mL), amphotericin B (25 μg/mL) and incubated in a stove at 37 °C with 5% CO_2_. Cells were subcultured (5–7 days), using 0.9% saline to wash them and 0.25% trypsin to disintegrate them from the vial. After disruption, these cells were centrifuged at 1000 rpm for 10 min at 10 °C, resuspended in complete DMEM medium (supplemented with FBS), and cell counted in a Neubauer’s chamber. 

The sub-cultured third to eighth passage fibroblasts were inoculated into 96-well microplates at a density of 0.5 × 10^5^ cells/well. They were then incubated at 37 °C with 5% CO_2_. After 24 h, 20 µL of different dilutions of each sample were added to the wells of the plate containing the cells in medium not supplemented with SBF (incomplete medium) and incubated. Twenty-four hours post-treatment, the medium was withdrawn, cells were washed with saline and 20 µL of resazurin (Sigma-Aldrich) 0.01% *w/v* in deionized H_2_O was added to each well containing 180 µL of DMEM medium supplemented with 10% Of SFB. The plates were then incubated for 4 h at 37 °C and fluorescence was measured at 540 and 590 nm for excitation and emission, respectively [59]. Cell viability was expressed as a percentage of viable cells when compared to the control group without treatment.

### 4.10. Statistical Analysis

GraphPad Prism software (GraphPad Software, Inc., La Jolla, CA, USA) was employed for the statistical analysis with a confidence level of 95%. Parametric statistical analyses were conducted with one-way ANOVA followed by Tukey’s multiple comparison test for total phenols and zeta potential. For the ion test the statistical analyses was Dunn’s multiple comparison test.

## 5. Conclusions

In light of the results obtained and the limitations of the present study, it was concluded that the use of pomegranate peel extract showed it to be an efficient reducing agent for the production of silver nanoparticles. Moreover, the antimicrobial potential and the low cytotoxicity demonstrated by green-silver nanoparticles have stimulated the search for improvements in the bio-nanotechnology field. Furthermore, the anti-inflammatory and antioxidant properties of pomegranate have encouraged further studies to use nanosystems with future application in prophylaxis or treatment of biofilm-dependent diseases.

## Figures and Tables

**Table 1:** Values of the silver ionic reduction and zeta potential for green and chemical silver nanoparticle formulations in different periods.

Time	Silver Nanoparticles Green Formulation			Silver Nanoparticles Chemical Formulation		
	µgAg^+^/mL	% of Reduction	Zeta Potential	µgAg^+^/mL	% of Reduction	Zeta Potential
T0	0.249	99.93%	−73.7 ± 6.49	1.769	68.15%	−78.2 ± 3.06
T7	0.178	99.95%	−68.3 ± 4.92	1.927	65.31%	−72.9 ± 3.10
T14	0.220	99.94%	−72.8 ± 6.49	1.543	72.22%	−85.5 ± 3.36
T28	0.186	99.95%	−68.6 ± 5.62	1.846	66.77%	−76.5 ± 4.05

**Table 2:** Silver ion concentration (µgAg^+^/mL) and percentage of silver ions reduction after the reactions, AgNP percentage, and values of minimum inhibitory concentration (MIC) of silver nanoparticles and pomegranate peel extract found for *Staphylococcus aureus and Candida albicans.*

Samples	Silver Ions Concentration	Silver Ions Remaining %	Ag NP %	MIC (µg/mL)	
				*S. aureus*	*C. albicans*
Control *	10,303.26	95.52	4.48	4.13	4.59
Pomegranate peel extract	-	-	-	391	781
Silver nanoparticles green	10.89	0.11	99.89	67.50	68.75
Silver nanoparticles chemical	130.40	1.21	98.79	0.50	0.25
Pomegranate peel extract formulation	-	-	-	0.37	0.18
Silver nanoparticles green formulation	0.249	0.01	99.99	0.26	16.87
Silver nanoparticles chemical formulation	1.769	31.85	68.15	0.56	1.12

* Control = Carboxymethylcellulose, propylene glycol, silver nitrate.
